# Developing survey weights to ensure representativeness in a national, matched cohort study: results from the children and young people with Long Covid (CLoCk) study

**DOI:** 10.1186/s12874-024-02219-0

**Published:** 2024-06-20

**Authors:** Natalia K Rojas, Bianca L De Stavola, Tom Norris, Mario Cortina-Borja, Manjula D Nugawela, Dougal Hargreaves, Emma Dalrymple, Kelsey McOwat, Ruth Simmons, Terence Stephenson, Roz Shafran, CLoCk Consortium, Snehal M Pinto Pereira

**Affiliations:** 1 Division of Surgery & Interventional Science, Faculty of Medical Sciences, University College London, London, WC1E 6BT UK; 2 UCL Great Ormond Street Institute of Child Health, 30 Guilford Street, London, WC1N 1EH UK; 3 Mohn Centre for Children’s Health & Wellbeing, School of Public Health, Imperial College London, London, UK; 4 Immunisation Department, Health Security Agency, 61 Colindale Avenue, London, NW9 5EQ UK

**Keywords:** Long covid, Survey weights, Children and young people, Representativeness, Matched cohort study, Selection bias, Attrition, Loss to follow-up, Post Covid Condition

## Abstract

**Background:**

Findings from studies assessing Long Covid in children and young people (CYP) need to be assessed in light of their methodological limitations. For example, if non-response and/or attrition over time systematically differ by sub-groups of CYP, findings could be biased and any generalisation limited. The present study aimed to (i) construct survey weights for the Children and young people with Long Covid (CLoCk) study, and (ii) apply them to published CLoCk findings showing the prevalence of shortness of breath and tiredness increased over time from baseline to 12-months post-baseline in both SARS-CoV-2 Positive and Negative CYP.

**Methods:**

Logistic regression models were fitted to compute the probability of (i) Responding given envisioned to take part, (ii) Responding timely given responded, and (iii) (Re)infection given timely response. Response, timely response and (re)infection weights were generated as the reciprocal of the corresponding probability, with an overall ‘envisioned population’ survey weight derived as the product of these weights. Survey weights were trimmed, and an interactive tool developed to re-calibrate target population survey weights to the general population using data from the 2021 UK Census.

**Results:**

Flexible survey weights for the CLoCk study were successfully developed. In the illustrative example, re-weighted results (when accounting for selection in response, attrition, and (re)infection) were consistent with published findings.

**Conclusions:**

Flexible survey weights to address potential bias and selection issues were created for and used in the CLoCk study. Previously reported prospective findings from CLoCk are generalisable to the wider population of CYP in England. This study highlights the importance of considering selection into a sample and attrition over time when considering generalisability of findings.

**Supplementary Information:**

The online version contains supplementary material available at 10.1186/s12874-024-02219-0.

## Background

By March 2022, most children and young people (CYP) in the United Kingdom (UK) appeared to have been exposed to SARS-CoV-2, with antibodies found in 82% and 99% of primary and secondary school aged pupils, respectively [1]. Given the scale of infection, a substantial number could develop symptoms of Long Covid (also referred to as Post Covid Condition). Long Covid in CYP can be defined as the presence of one or more impairing, persisting, physical symptom(s) lasting 12 or more weeks after initial SARS-CoV-2 infection that may fluctuate or relapse, either continuing or developing post-infection [2]. Hence, it is important to study Long Covid, particularly given its potential impact on healthcare systems and need for planning.

Systematic reviews demonstrate that common symptoms of Long Covid in CYP at 3 months post-testing/infection include fatigue, insomnia, loss of smell, and headaches [3]. The Long Covid (CLoCk) study, is the largest matched cohort study of Long Covid in CYP in the world [4]. Based in England, CLoCk collected data on over 30,000 CYP testing positive and negative between September 2020 and March 2021 over a two-year period. CLoCk followed 6,804 CYP 3 months after a SARS-CoV-2 PCR-test and found over half of CYP testing negative and 67% of those testing positive reported at least one symptom 3-months post-testing [5]. The most common symptoms amongst test positives were tiredness (39%), headache (23%) and shortness of breath (23%), with test negatives reporting mainly tiredness (24%) and headache (14%). Results from this, and all other studies, need to be assessed against their methodological limitations, two of which are considered here. First, response rates to study invitation are generally low, for example, the response rate at the 3-months post-testing sweep of the CLoCk study was 13.4% [5]. Similarly, the UK Office for National Statistics’ [6] COVID-19 infection survey had a response rate of 12%. Second, all longitudinal studies suffer from attrition over time [7] which is typically more pronounced in studies with longer follow-up periods [8].

If non-response and attrition over time systematically differ by sub-groups in the envisioned population, findings could be biased and attempts to generalise findings to the wider population limited [9–11]. For example, those with particular characteristics (e.g., older, females and from specific ethnicities) are more likely to positively respond to study invitation [12]. Reasons for attrition over time include study withdrawal, individuals becoming uncontactable [e.g., due to change in contact details; 13] or lacking motivation to continue participating. Indeed, both initial non-respondents and those lost to follow-up are often socioeconomically disadvantaged and less healthy [14]. With studies on Long Covid, particularly those comparing test-positives to test-negatives, an additional source of bias could exist. For example, within the CLoCk study, to isolate the effect of Long Covid from that of living through a pandemic, researchers originally excluded from the analytic sample those (re)infected, that is, test-negatives who subsequently tested positive and test-positive CYP who were subsequently reinfected [15]. This criterion yields a cohort of CYP who, as per the data available, appear to have either (i) always tested negative, or (ii) tested positive only once. However, these CYP may not be representative of the larger population of CYP in England. One well-established method to assess the impact of potential bias due to non-response, attrition and sample selection is weighting, that is, emphasising the contribution of some individuals over others in an analysis to reconstruct the target population and/or general population [9]. Such weighting methodology is appropriate when data are missing (due to non-response, attrition, and sample selection) at random [16], that is, the missingness is dependent on fully observed characteristics such as sex, age, socioeconomic disadvantage and health status. Yet, this powerful statistical technique to address potential selection biases has been underutilised in epidemiological research [9].

In this manuscript we construct weights for the CLoCk study [17] and, as an illustrative example, apply them to published findings showing the overall prevalence of shortness of breath and tiredness increases in CYP from baseline (i.e., at the time of their index PCR test) to 12-months post-baseline [15]. Specifically, to assess the robustness of conclusions drawn from CLoCk data about Long Covid’s symptomatology and trajectory in CYP, the present study aims to (i) create weights for the CLoCk study at its data collection sweeps 3-, 6- and 12-months post-index PCR-test, and (ii) apply developed weights to the analysis of shortness of breath and tiredness over a 12-month period to determine whether accounting for any biases in response, attrition or (re)infection affects published results.

## Methods

**Fig. 1:**
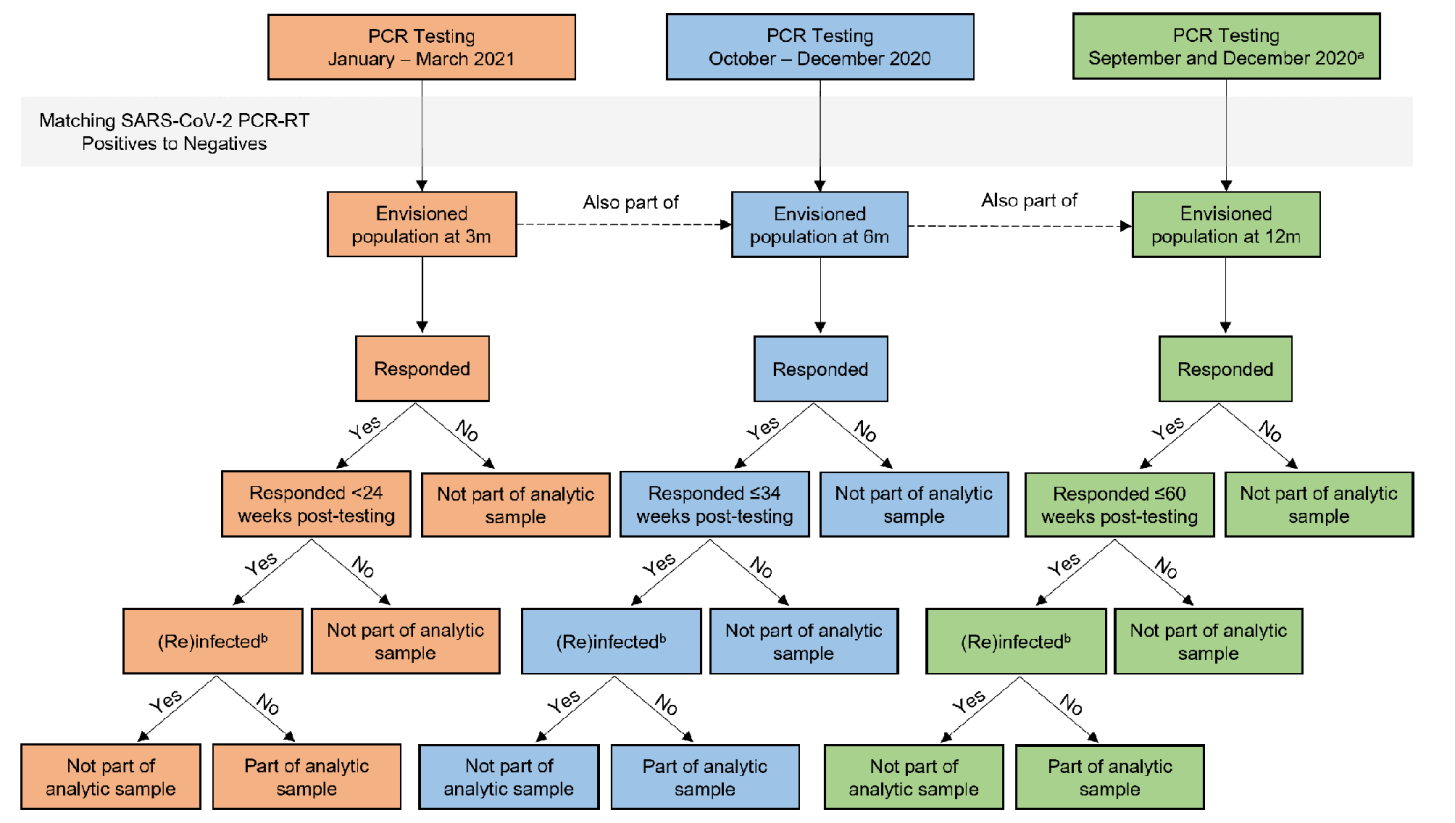
Logic model for inclusion in the analytic sample at 3-, 6-, and 12-months ^a^ Initially, due to funding constraints, only a portion of those tested in December 2020 were contacted to participate at 6 months. Hence, some children and young people tested in December 2020 provided both 6- and 12- month data, whereas others only 12-month data ^b^ Determined through self-report and UKHSA data. (Re)infected refers to (i) a SARS-CoV-2 Negative subsequently testing positive, or (ii) a SARS-CoV-2 Positive testing positive again

**Fig. 2:**
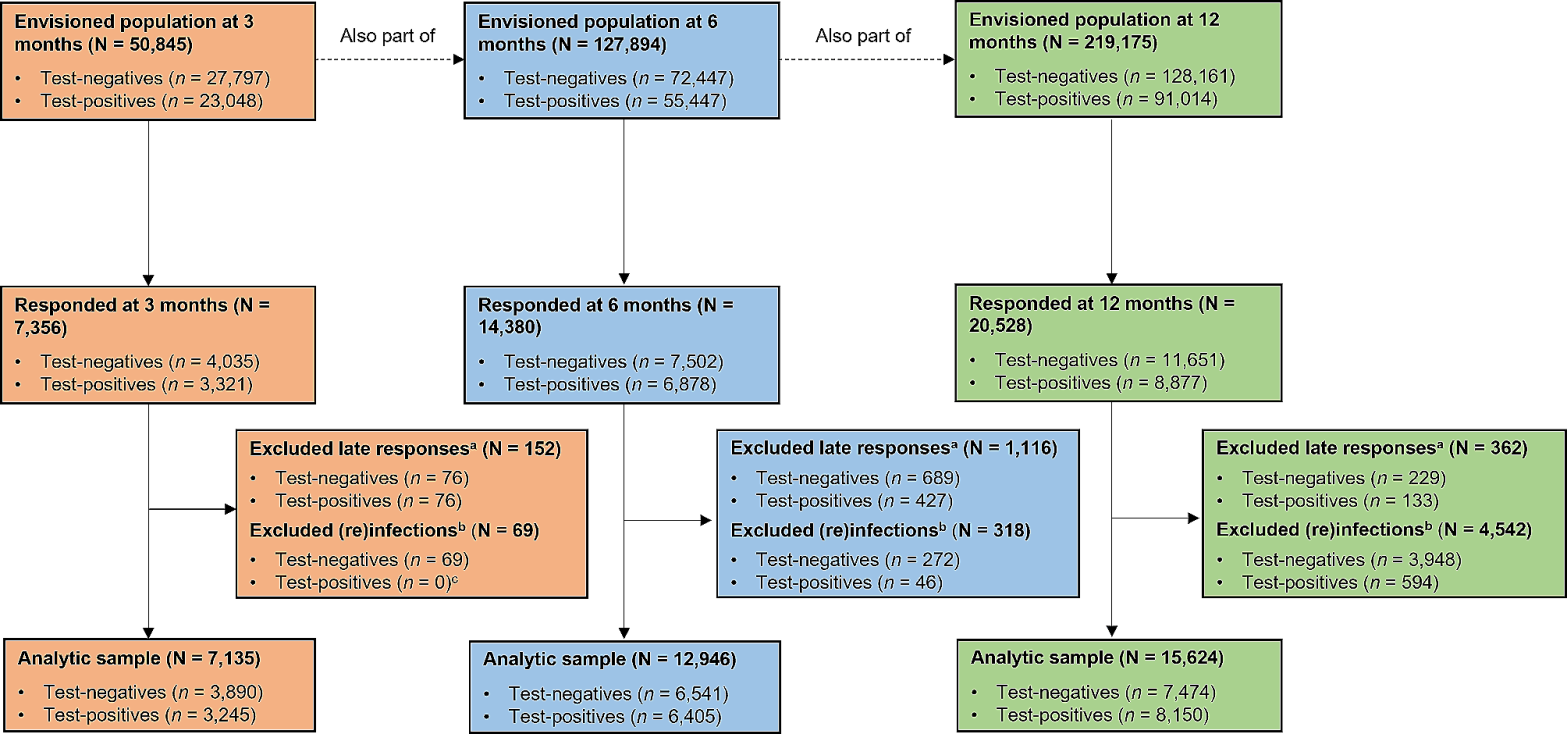
Flow diagram of participants at 3-, 6-, and 12 months ^a^ Determined using the following cut off points: < 24 weeks post-testing for the 3-month questionnaire; ≤ 34 weeks post-testing for the 6-month questionnaire; ≤ 60 weeks post-testing for the 12-month questionnaire ^b^ Determined through self-report and UKHSA data. (Re)infected refers to (i) a SARS-CoV-2 Negative subsequently testing positive, or (ii) a SARS-CoV-2 Positive testing positive again ^c^ By definition of a COVID positive episode [19], a test-positive person cannot be reinfected by 3 months

The CLoCk study identified 219,175 CYP (91,014 SARS-CoV-2 Positive and 128,161 SARS-CoV-2 Negative) who had a SARS-CoV-2 PCR-test between September 2020 and March 2021 through the UK Health Security Agency’s (UKHSA) database containing the outcomes of all such tests. At study invitation, test-positives were matched to test-negatives on age, sex, region of residence and month of test. Consenting SARS-CoV-2 Positive and Negative CYP complete a questionnaire about their mental and physical health 3-, 6-, 12- and 24-months post-index PCR-test [4]. Of note, the sweeps of data collection depend on the CYP’s month of test, with 3-, 6-, 12-, and 24-month data available for some (tested in January-March 2021), while for others only 6-, 12-, and 24-month (tested in October-December 2020), or 12- and 24-month (tested in September 2020 and an additional cohort from December 2020) data were collected. This manuscript is based on all data collected for the 3-, 6-, and 12-month timepoints. The analytic samples for previous CLoCk publications [5, 15, 18] were such that: (i) CYP must have responded within a pre-specified timeframe (i.e., < 24, ≤34, and ≤ 60 weeks post-testing for the 3-, 6-, and 12-month questionnaires, respectively) and (ii) Initial SARS-CoV-2 Negative CYP must have never reported a positive test, with initial SARS-CoV-2 Positive CYP never reporting being reinfected. The latter requirement was determined using a combination of self-report and UKHSA held data. See Figs. 1 and 2 for exclusion criteria at each stage and participant flow.

Research ethics approval was granted by the Yorkshire and The Humber—South Yorkshire Research Ethics Committee (REC reference: 21/YH/0060; IRAS project ID:293,495).

### Measures

Index COVID status, age, sex and region were determined from data held at UKHSA. Socioeconomic status was proxied using the Index of Multiple Deprivation (IMD), obtained using CYP’s lower super output area (i.e., small local area level-based geographic hierarchy), where higher values are indicative of lower deprivation [20]. Ethnicity was self-reported and collected at registration. Current (i.e., at time of questionnaire completion) health, current loneliness, and number of symptoms being experienced, including tiredness and shortness of breath, [out of a possible 21, consistent with the ISARIC Paediatric Working Group; 5] were self-reported at each data collection sweep. Similarly, standardised measures were collected, including the: Short Warwick and Edinburgh Mental Wellbeing Scale [SWEMWS; 21]; EuroQol Visual Analogue Scale [EQ-VAS; 22], EQ-5D-Y [23], Strengths and Difficulties Questionnaire [SDQ; 24], UCLA Loneliness Scale [25], and Chalder Fatigue Scale [CFS; 26]. See Additional File 1: Table 1 for further information.

For each data collection sweep, three indicator variables were created:

- Responding given envisioned to take part (Yes/No): If participants completed the whole questionnaire.
- Responding timely given responded (Yes/No): If participants who responded, responded to the questionnaire < 24 weeks post-testing (3-month questionnaire); ≤ 34 weeks post-testing (6-month questionnaire) and ≤ 60 weeks post-testing (12-month questionnaire).
- (Re)infected given timely response (Yes/No): ‘Yes’ indicates, among those responding timely, SARS-CoV-2 index-test Positives that were reinfected and SARS-CoV-2 index-test Negatives that subsequently tested positive. ‘No’ indicates, among those responding timely, initial SARS-CoV-2 Positives that never report another positive test and initial SARS-CoV-2 Negatives that never report a positive test. A combination of the UKHSA’s testing data and self-reported information on having ever tested positive was used to generate this.

In total nine indicator variables were created: three at each data collection sweep.

### Analysis

Analyses were conducted using Stata v17 [27].

#### Weight generation

**Fig. 3:**
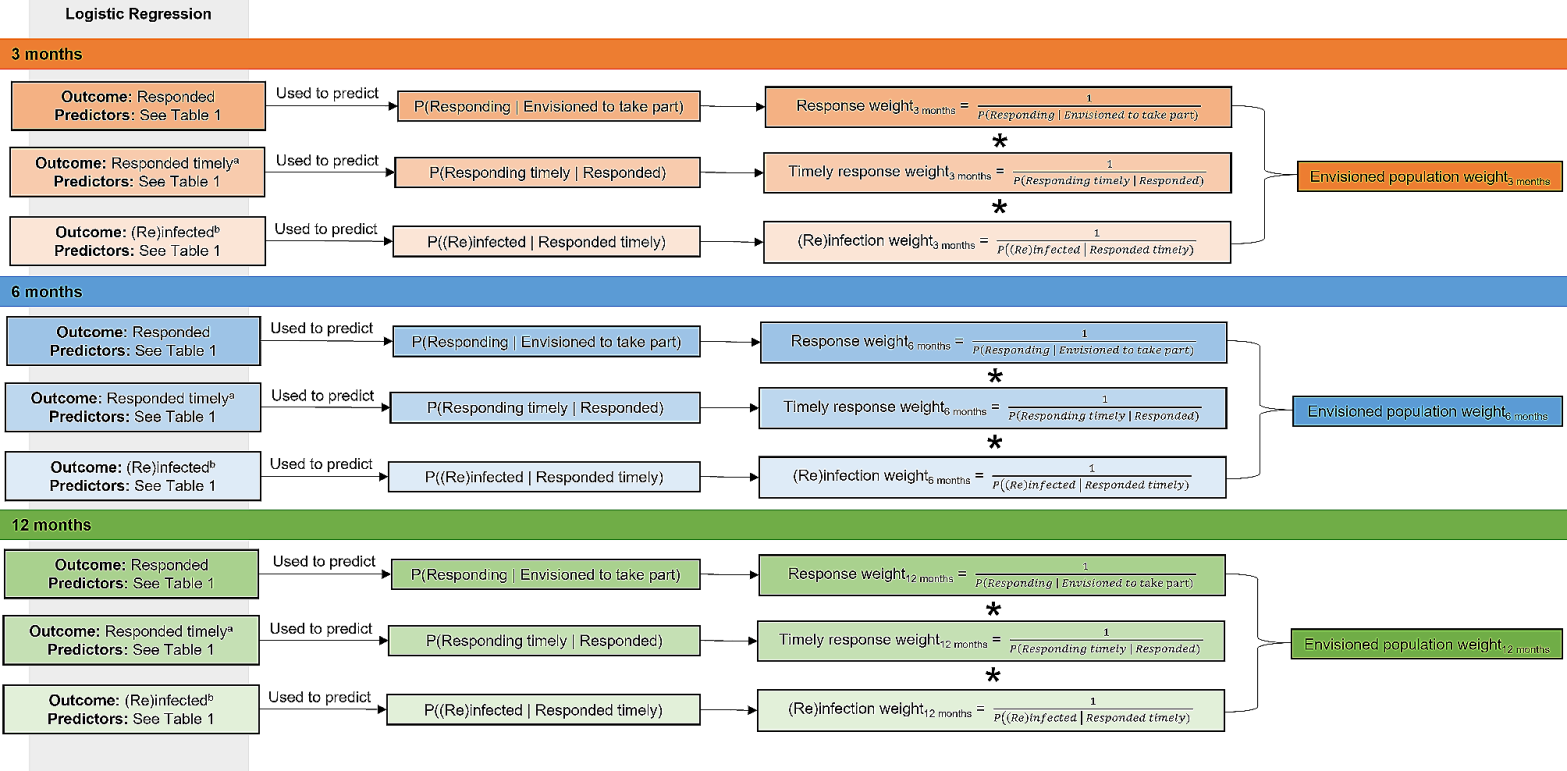
Steps in weight generation ^a^ Determined using the following cut off points: < 24 weeks post-testing for the 3-month questionnaire; ≤ 34 weeks post-testing for the 6-month questionnaire; ≤ 60 weeks post-testing for the 12-month questionnaire ^b^ Determined through self-report and UKHSA data. (Re)infected refers to (i) a SARS-CoV-2 Negative subsequently testing positive, or (ii) a SARS-CoV-2 Positive testing positive again

At each data collection sweep and corresponding to the three indicator variables created (as described above), three ‘mini’ survey weights were generated to account for CYP being lost either due to (i) non-response, (ii) responding after the established cut-off points or (iii) (re)infection with SARS-CoV-2. A fourth, combined ‘envisioned population’ weight was created which accounted for loss in the analytic sample due to all three factors. These four survey weights (three ‘mini’ survey weights and one ‘envisioned population’ weight) were generated for each data collection sweep, (i.e., 3-, 6- and 12-months post-SARS-CoV-2 test), see Fig. 3 for details.

**Fig. 4:**
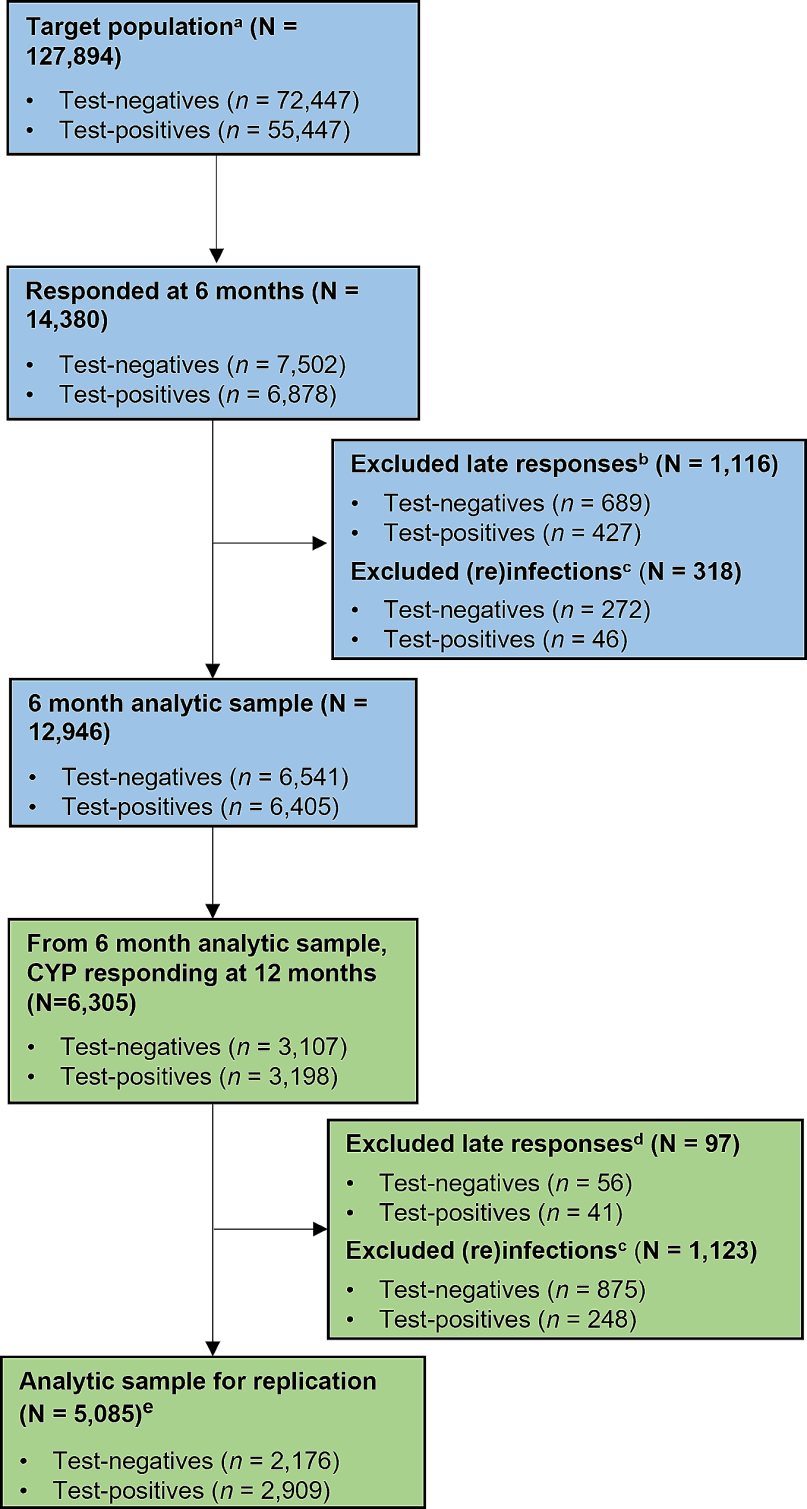
Participant flow in the published CLoCk study [15] to be replicated ^a^ Here, the target population is all children and young people that could have taken part at 6 months ^b^ A late response at 6 months is defined as not responding ≤ 34 weeks post-testing ^c^ Determined through self-report and UKHSA data. (Re)infected refers to (i) a SARS-CoV-2 Negative subsequently testing positive, or (ii) a SARS-CoV-2 Positive testing positive again ^d^ A late response at 12 months is defined as not responding ≤ 60 weeks post-testing ^e^ Of these, 1,826 children and young people registered at 3 months (806 SARS-CoV-2 Negative and 1,020 SARS-CoV-2 Positive)

Here, the term ‘envisioned’ population refers to all CYP that could have taken part at the relevant time point (i.e., it is the maximum number of CYP that could provide data at a specific time point and was 50,845, 127,894 and 219,175 at 3-, 6-, and 12-months respectively). The ‘target’ population varies depending on the specific research question. For example, in the illustrative example described below, the target population is all CYP that could have taken part at 6 months (i.e., *N* = 127,894; see Fig. 4).

The three ‘mini’ survey weights were calculated for (i) response given envisioned to take part, (ii) timely response given response, and (iii) (re)infection given timely response. Each ‘mini’ survey weight was calculated as the reciprocal of its corresponding conditional probability (Fig. 3). These conditional probabilities were computed using logistic regression (described below).

For the logistic regression of responding given envisioned to take part, all available data (held at UKHSA for study-design matching) and pair-wise interactions were considered as explanatory variables. For the logistic regressions of (i) responding timely given responded and (ii) (re)infected given timely response, questionnaire data was also available for use as predictors. Forward (*p <* 0.157) and backward (*p <* 0.200) stepwise selection processes were used to refine models used to predict these probabilities with cut-offs selected as per recommendations [28]. Our weighting approach is appropriate when data are missing at random [16]. In an attempt to ensure this assumption is valid we included sex, age, region, index COVID Status and IMD in all but one (see below) of the logistic regression models. Of these, age and IMD were continuous variables, while the others were categorical. We determined the appropriate functional form for the relationship between age/IMD and the log odds of the probability of the (three) outcomes by modelling the relationship (i) linearly, (ii) categorically (age: 11–13, 14–15, 16–17 years; IMD deciles, 1–5), (iii) with linear and quadratic terms and (iv) using fractional polynomials with up to two degrees. The functional forms with the lowest Akaike’s information criterion (i.e., the best fitting model) were used in our subsequent models. Importantly, index COVID Status was excluded as a predictor of the probability of being (re)infected given CYP responded timely at 3 months. This is because, by definition of a COVID positive episode [19], once a person tests positive, they would only be considered to be reinfected should they test positive more than 3 months after the initial positive test. Table 1 summarises the variables included in each model to predict the three conditional probabilities at the three timepoints. When issues with variables perfectly predicting the outcome were encountered, relevant variables were dropped. This only happened at the 3-month time-point. The concordance statistic (*C)* was used to assess the predictive performance of the models: values 0.7 and 0.8 denoting good and strong performance, respectively, with a value of ≤ 0.5 indicating poor prediction [Table 1; 29, 30].

**Table 1:** Variables included in logistic regression models used to produce conditional probabilities for weight generation

Conditional probability	3 months post index PCR-test			6 months post index PCR-test			12 months post index PCR-test		
	Outcome	Predictors*	C-Statistic	Outcome	Predictors*	C-Statistic	Outcome	Predictors*	C-Statistic
Pr(Responding | Envisioned to take part)	Responding to 3-month questionnaire	Sex, Age (cubed and cubed multiplied by log of age), Region, Index COVID Status, IMD (squared and linear) and all 2-way interactions	0.6207	Responding to 6-month questionnaire	Sex, Age (cubed and cubed multiplied by log of age), Region, Index COVID Status, IMD (squared and linear) and all 2-way interactions	0.6281	Responding to 12-month questionnaire	Sex, Age (cubed and cubed multiplied by log of age), Region, Index COVID Status, IMD (to power of 0.5) and all 2-way interactions	0.6388
Pr(Responding timely | Responded)	Responding < 24 weeks post index-test	Sex, Age (Categorical: 11–13; 14–15; 16–17), Region, Index COVID Status, IMD (squared and linear), Vaccination Status, Current Physical Health, Current Loneliness	0.7301	Responding ≤ 34 weeks post index-test	Sex, Age (Categorical: 11–13; 14–15; 16–17), Region, Index COVID Status, IMD (square root and to power of -2), Ethnicity, Current EQ-VAS Score, Current number of symptoms, Vaccination Status	0.6666	Responding ≤ 60 weeks post index-test	Sex, Age (Linear), Region, Index COVID Status, IMD (Linear and squared), Ethnicity, Vaccination Status, Current CFS Score	0.6001
Pr((Re)infected | Responded timely)	(Re)infected by 3 months, given responded < 24 weeks post index-test	Sex, Age (Linear), IMD (squared and linear), Current SWEMWS Score, Current Physical Health, Current EQ-VAS, Current Number of Symptoms	0.7020	(Re)infected by 6 months, given responded ≤ 34 weeks post index-test	Sex, Age (cubed and cubed multiplied by log of age), Region, Index COVID Status, IMD (squared and linear), Vaccination Status, Current Number of Symptoms, Current SDQ, Current EQ-5D-Y Score	0.7702	(Re)infected by 12 months, given responded ≤ 60 weeks post index-test	Sex, Age (Linear), Region, Index COVID Status, IMD (squared and to power of -2), Ethnicity, Current Number of Symptoms, Current SDQ, Current EQ-5D-Y, Current Physical Health, Current EQ-VAS, Current Loneliness, Vaccination Status	0.7739

*Note*. For Pr(Responding timely | Responded) at 3 months, inclusion of ethnicity resulted in perfect prediction and was dropped. For Pr((Re)infected | Responded timely) at 3 months: inclusion of Region and Ethnicity resulted in perfect prediction and were thus dropped. Here, COVID Status was not included in the stepwise selection process as, by definition of a COVID positive episode [19], a test-positive person cannot be reinfected by 3 months

*See Additional File 1; Table 1 for more information on these variables and their handling in the present study

At each time-point, the envisioned population weight was calculated as the product of the three corresponding ‘mini’ survey weights. Taking the example of 3 months post-testing: to re-weight from the previously used analytic sample to the envisioned CLoCk population, the fourth created survey weight comprised the product of the following three survey weights: Response_3 months_, Timely response_3 months_, and (Re)infection_3 months_ (Fig. 3). The four survey weights at each time point (twelve in total) are flexible and can be combined as required, to create final survey weights to get to the target population as described in the illustrative example.

#### Weighting to the general population

Generated survey weights re-weight the analytic sample to the CLoCk envisioned population, that is, CYP invited to participate if they had a PCR-test within the pre-specified timepoints. However, as PCR testing varied by region and stage of the pandemic [31, 32], the envisioned population may not be fully representative of the general population of CYP in England. This is because, for example, not all CYP in England will have been able to access/complete a PCR-test. Hence, final survey weights used to get to the required target population were re-calibrated to the general population, using data on sex, age, and region from the 2021 UK Census [33]. To do this, ratios of the Census data to CLoCk data reweighted to the target population of interest were produced (see Additional File 2 for the interactive tool used to calculate these) with the final target population survey weights then multiplied by these ratios. See Additional File 2 for how this was done for the illustrative example below.

#### Weight trimming

All survey weights (i.e., each of the response given envisioned to take part, timely response given response, (re)infection given timely response, and the ‘envisioned population’ survey weights) were trimmed to reduce the likelihood of extremely large survey weights increasing variance [34]. This was done by reducing extreme survey weights to a cut-off defined as the median + *k* × interquartile range. *k* is typically either 3 or 4 [35]. In the present study we took a conservative approach and set *k* as 3. All survey weights were multiplied by a factor to re-calibrate back up to the original sum of weights [36]. When combining survey weights for the illustrative example below, untrimmed survey weights were initially used with the final survey weights trimmed.

#### Illustrative example: replicating published findings

**Fig. 5:**
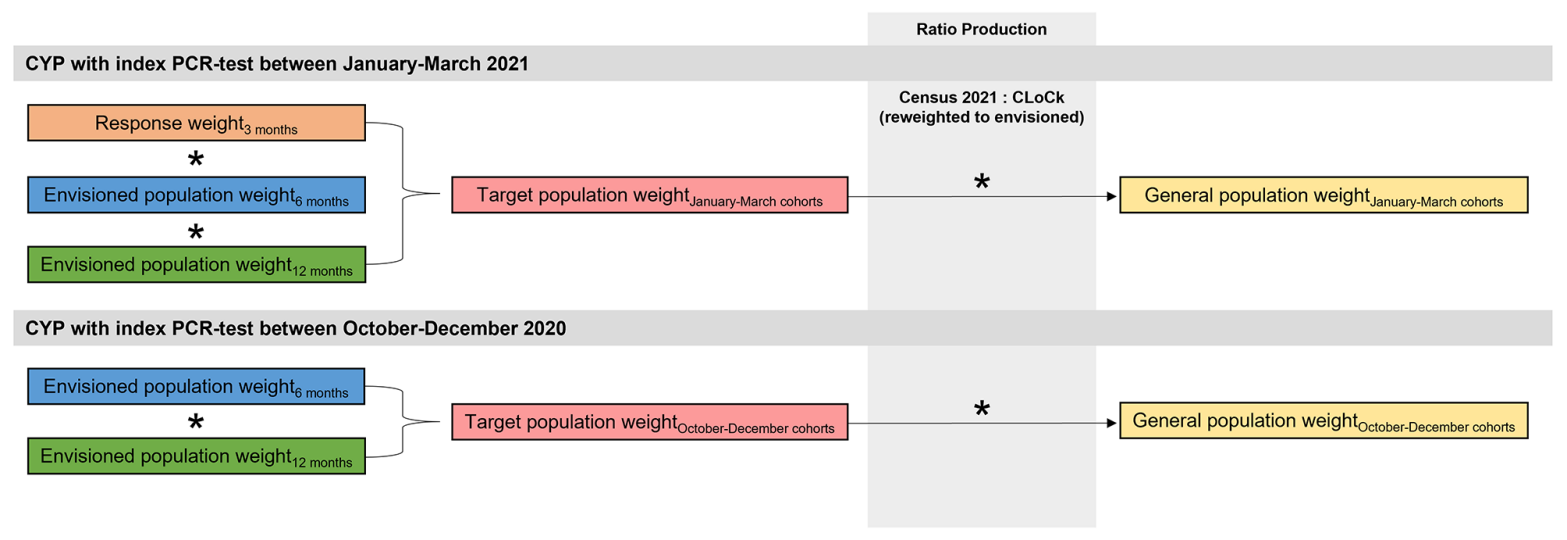
Steps taken to combine survey weights to replicate published CLoCk findings [15] *Note*. To be included in the analytic sample, children and young people must have provided information about their symptoms at the time of their PCR test (i.e., 0 months). This information is gathered at study enrolment meaning criteria for inclusion varied depending on month of index PCR-test. Children and young people with an index test in January, February and March 2021 must have responded to the 3-month questionnaire (to gather information about baseline symptoms) as well as meet the criteria for inclusion in the analytic samples at 6- and 12-months post-testing (i.e., responded, done so timely and not (re)infected). Children and young people with an index-test in October, November, and December 2020 only had to meet the criteria for inclusion in the analytic samples at 6- and 12-months

Findings from CLoCk show the overall prevalence of tiredness and shortness of breath are high in CYP at baseline (i.e., at the time of their index PCR test) and increase over time to 12 months [15]. Here we compare the prevalence of tiredness and shortness of breath over a 12-month period from a previous publication [15] to prevalences that were weighted to the (i) target, and (ii) general populations. We demonstrate how uncertainty around generated weights can be accounted for via bootstrapping (with 1000 replications) and supply illustrative code for this (Additional File 1: Text 1). To be included in the published analytic sample (*n* = 5,085), CYP first registering in January-March 2021 must have completed their 3-month questionnaire (to provide information about their symptoms at the time of their PCR-test, i.e., at baseline), and be in the analytic sample at 6- and 12-months. For those registering in October-December 2020, they must meet the requirements to be included in the analytic samples at both 6- and 12-months (see Fig. 1 for cohort breakdown and Fig. 4 for participant flow for this example). Therefore, longitudinal weights were created by combining the survey weights as detailed in Fig. 5 and further illustrated in the bootstrap example in Text 1 (Additional File 1).

## Results

At the 3-month sweep, 7,135 CYP were included in the analytic sample, constituting 14% of the envisioned population at that time-point (*N* = 50,845, Table 2; Fig. 2). The analytic sample at 6 months (*n* = 12,946) comprised 10% of the envisioned population (*N* = 127,894); at 12-months, 15,624 were included in the analytic sample, forming 7% of the 12-month envisioned population (*N* = 219,175). Overall, 31,012 CYP completed at least one questionnaire, with 42,264 questionnaires completed. CYP in the analytic samples at 3-, 6-, and 12-months completed the questionnaire at a median of 14.9 (IQR: 13.1–18.9), 27.9 (IQR: 26.3–29.7), and 52.7 (IQR: 51.3–54.9) weeks post-testing, respectively. Compared to the envisioned population, CYP in the analytic samples were older, female and from less deprived areas (Table 2).

**Table 2:** Characteristics of the 3-, 6-, and 12-month envisioned and analytic populations

	3 months		6 months		12 months	
	Envisioned population (*N* = 50,845)	Analytic sample (*N* = 7,135)	Envisioned population^a^ (*N* = 127,894)	Analytic sample (*N* = 12,946)	Envisioned population^b^ (*N* = 219,175)	Analytic sample (*N* = 15,624)
*Negative SARS-CoV-2 test*	*27,797*	*3,890*	*72,447*	*6,541*	*128,161*	*7,474*
Sex						
Male	12,678 (45.6)	1,443 (37.1)	33,941 (46.9)	2,430 (37.2)	60,967 (47.6)	2,830 (37.9)
Female	15,119 (54.4)	2,447 (62.9)	38,506 (53.2)	4,111 (62.9)	67,194 (52.4)	4,644 (62.1)
Age (Years)						
11–14	12,689 (45.7)	1,689 (43.4)	34,832 (48.1)	2,814 (43.0)	65,951 (51.5)	3,481 (46.6)
15–17	15,108 (54.4)	2,201 (56.6)	37,615 (51.9)	3,727 (57.0)	62,210 (48.5)	3,993 (53.4)
Region (England)						
East Midlands	2132 (7.7)	357 (9.2)	6,231 (8.6)	709 (10.8)	7,861 (6.1)	401 (5.4)
East of England	4277 (15.4)	646 (16.6)	7,272 (10.0)	742 (11.3)	24,919 (19.4)	1,906 (25.5)
London	5356 (19.3)	649 (16.7)	10,178 (14.1)	824 (12.6)	27,156 (21.2)	1,832 (24.5)
North East England	925 (3.3)	127 (3.3)	4,098 (5.7)	379 (5.8)	4,825 (3.8)	199 (2.7)
North West England	3816 (13.7)	441 (11.3)	13,590 (18.8)	920 (14.1)	17,950 (14.0)	704 (9.4)
South East England	4262 (15.3)	655 (16.8)	8,923 (12.3)	890 (13.6)	18,251 (14.2)	1,236 (16.5)
South West England	1554 (5.6)	300 (7.7)	4,013 (5.5)	489 (7.5)	4,563 (3.6)	240 (3.2)
West Midlands	3414 (12.3)	455 (11.7)	9,748 (13.5)	877 (13.4)	12,881 (10.1)	600 (8.0)
Yorkshire and the Humber	2061 (7.4)	260 (6.7)	8,394 (11.6)	711 (10.9)	9,755 (7.6)	356 (4.8)
IMD quintile^c^						
1 (most deprived)	8,097 (29.1)	784 (20.2)	21,583 (29.8)	1,286 (19.7)	31,116 (24.3)	1,121 (15.0)
2	6,299 (22.7)	761 (19.6)	14,735 (20.3)	1,175 (18.0)	25,744 (20.1)	1,306 (17.5)
3	4,978 (17.9)	763 (19.6)	12,546 (17.3)	1,219 (18.6)	23,423 (18.3)	1,435 (19.2)
4	4,439 (16.0)	751 (19.3)	12,063 (16.7)	1,370 (20.9)	23,725 (18.5)	1,711 (22.9)
5 (least deprived)	3,984 (14.3)	831 (21.4)	11,520 (15.9)	1,491 (22.8)	24,153 (18.9)	1,901 (25.4)
*Positive SARS-CoV-2 test*	*23,048*	*3,245*	*55,447*	*6,405*	*91,014*	*8,150*
Sex						
Male	10,636 (46.2)	1,201 (37.0)	26,004 (46.9)	2,413 (37.7)	42,972 (46.2)	3,072 (37.7)
Female	12,412 (53.9)	2,044 (63.0)	29,443 (53.1)	3,992 (62.3)	48,042 (52.8)	5,078 (62.3)
Age (Years)						
11–14	10,651 (46.2)	1,345 (41.5)	26,757 (48.3)	2,757 (43.0)	46,106 (50.7)	3,938 (48.3)
15–17	12,397 (53.8)	1,900 (58.6)	28,690 (51.7)	3,648 (57.0)	44.908 (49.3)	4,212 (51.7)
Region (England)						
East Midlands	1815 (7.9)	316 (9.7)	4,771 (8.6)	643 (10.0)	6,248 (6.9)	522 (6.4)
East of England	3392 (14.7)	483 (14.9)	5,546 (10.0)	649 (10.1)	13,982 (15.4)	1,567 (19.2)
London	4412 (19.1)	529 (16.3)	7,950 (14.3)	725 (11.3)	19,144 (21.0)	1,815 (22.3)
North East England	819 (3.6)	115 (3.5)	3,079 (5.6)	407 (6.4)	3,788 (4.2)	289 (3.6)
North West England	3235 (14.0)	396 (12.2)	10,363 (18.7)	981 (15.3)	13,339 (14.7)	878 (10.8)
South East England	3496 (15.2)	518 (16.0)	6,816 (12.3)	885 (13.8)	13,316 (14.6)	1,448 (17.8)
South West England	1238 (5.4)	248 (7.6)	2,934 (5.3)	498 (7.8)	3,576 (3.9)	358 (4.4)
West Midlands	2854 (12.4)	404 (12.5)	7,386 (13.3)	846 (13.2)	9,800 (10.8)	742 (9.1)
Yorkshire and the Humber	1787 (7.8)	236 (7.3)	6,602 (11.9)	771 (12.0)	7,831 (8.6)	531 (6.5)
IMD quintile^c^						
1 (most deprived)	6,732 (29.2)	686 (21.1)	16,498 (29.8)	1,267 (19.8)	22,963 (25.2)	1,192 (14.6)
2	5,198 (22.6)	660 (20.3)	11,528 (20.8)	1,168 (18.2)	19,013 (20.9)	1,455 (17.9)
3	4,159 (18.0)	603 (18.6)	9,589 (17.3)	1,120 (17.5)	16,453 (18.1)	1,527 (18.7)
4	3,679 (16.0)	625 (19.3)	9,112 (16.4)	1,340 (20.9)	16,271 (17.9)	1,797 (22.1)
5 (least deprived)	3,280 (14.2)	671 (20.7)	8,720 (15.7)	1,510 (23.6)	16,314 (17.9)	2,179 (26.7)

*Note*. Data are *n* (%). As rounded to 1 decimal point, percentages may sometimes add up to just above (100.1) or below (99.9) the expected 100%. Analytic samples contained only CYP (i) responding within a pre-specified timeframe (i.e., < 24, ≤34, and ≤ 60 weeks post-testing for the 3-, 6-, and 12-month questionnaires, respectively) and (ii) remaining negative (for initial SARS-CoV-2 negatives) and not reinfected (for initial SARS-CoV-2 positives). IMD = Index of Multiple Deprivation. ^a^ Includes envisioned population at 3 months; ^b^ Includes envisioned population at 3- and 6-months ^c^ IMD quintile 1 represents most deprived and quintile 5 represents least deprived

### Weight generation

The *C* statistics for all required conditional probabilities varied between 0.60 (responding timely given responded at 12 months) to 0.77 ((re)infected given timely response at 12-months and 6-months, see Table 1). Table 3 displays the survey weights generated for each data collection sweep along with the relevant *Ns*, medians, and interquartile ranges.

**Table 3:** Survey weights generated for each data collection sweep (*N*, Median, and Interquartile Range [IQR])

Data collection sweep	Survey weights	N	Median	IQR
**3 months**	Response	50,845	7.38	5.59–9.80
	Trimmed Response	50,845	7.38	5.59–9.80
	Timely Response	7,345	1.01	1.01–1.02
	Trimmed Timely Response	7,345	1.02	1.01–1.03
	(Re)infection	7,204	1.01	1.00-1.01
	Trimmed (Re)infection	7,204	1.01	1.01–1.01
	Envisioned population	7,124	6.52	5.04–8.60
	Trimmed envisioned population	7,124	6.52	5.04–8.60
**6 months**	Response	127,894	9.52	7.09–12.98
	Trimmed Response	127,894	9.52	7.09–12.98
	Timely Response	14,356	1.07	1.05–1.11
	Trimmed Timely Response	14,356	3.40	3.33–3.53
	(Re)infection	13,242	1.02	1.01–1.03
	Trimmed (Re)infection	13,242	1.02	1.01–1.03
	Envisioned population	12,926	8.74	6.64–11.89
	Trimmed envisioned population	12,926	8.77	6.67–11.94
**12 months**	Response	219,175	11.19	8.20-16.38
	Trimmed Response	219,175	11.19	8.20-16.38
	Timely Response	20,490	1.02	1.01–1.02
	Trimmed Timely Response	20,490	1.02	1.01–1.02
	(Re)infection	20,124	1.28	1.06–1.51
	Trimmed (Re)infection	20,124	1.28	1.06–1.51
	Envisioned population	15,593	11.70	8.64–16.53
	Trimmed envisioned population	15,593	11.97	8.84–16.91

*Note*. The number of survey weights generated differ from the *N*s in Fig. 2 as some CYP had missing data on the variables included within the logistic regression models used to generate the survey weights. Therefore, in some instances, not all eligible CYP were assigned a survey weight

### Re-weighting published findings

**Fig. 6:**
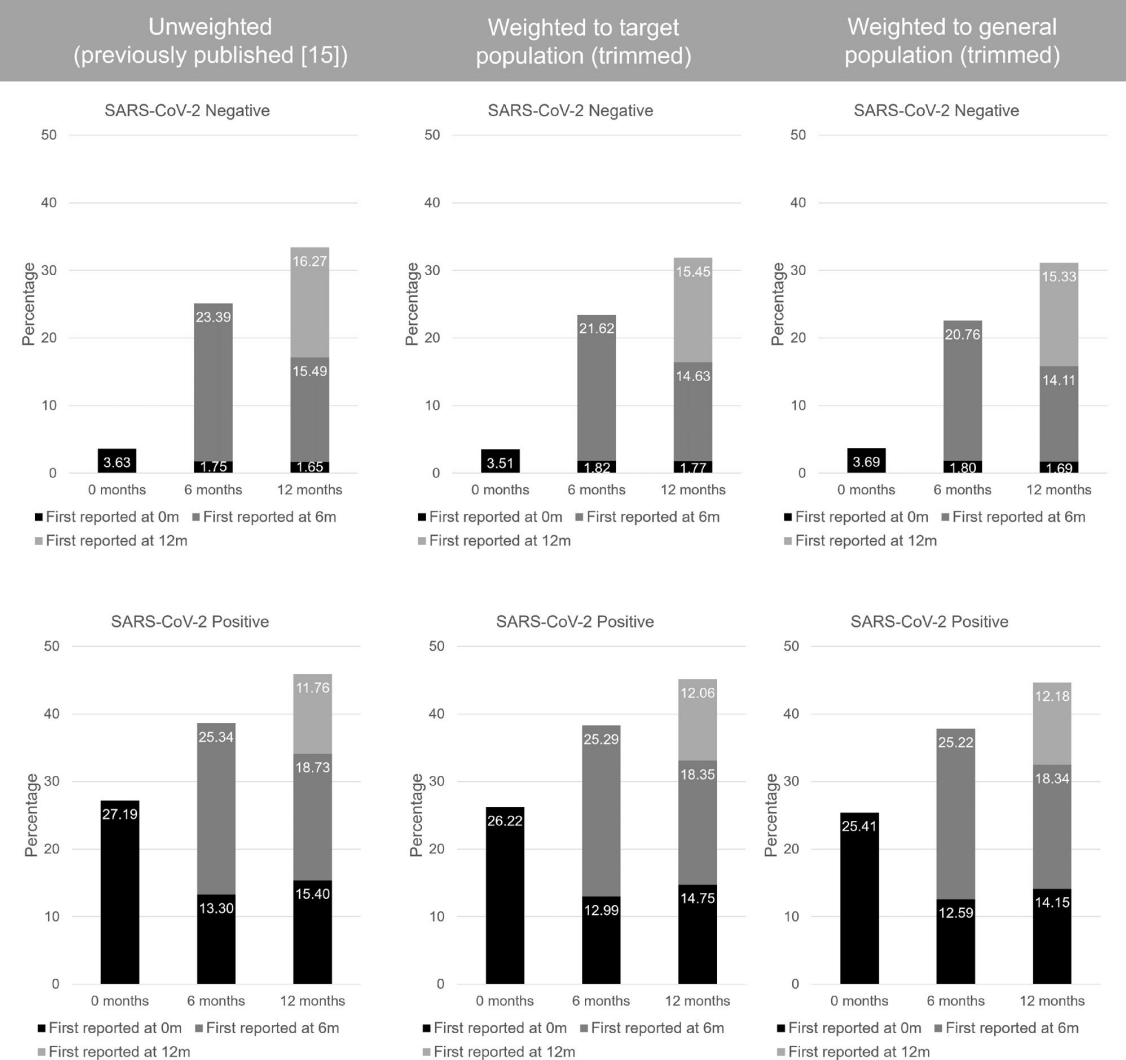
Weighted (trimmed) and unweighted tiredness prevalences 0-12-months post-index PCR-test by time of first report

**Fig. 7:**
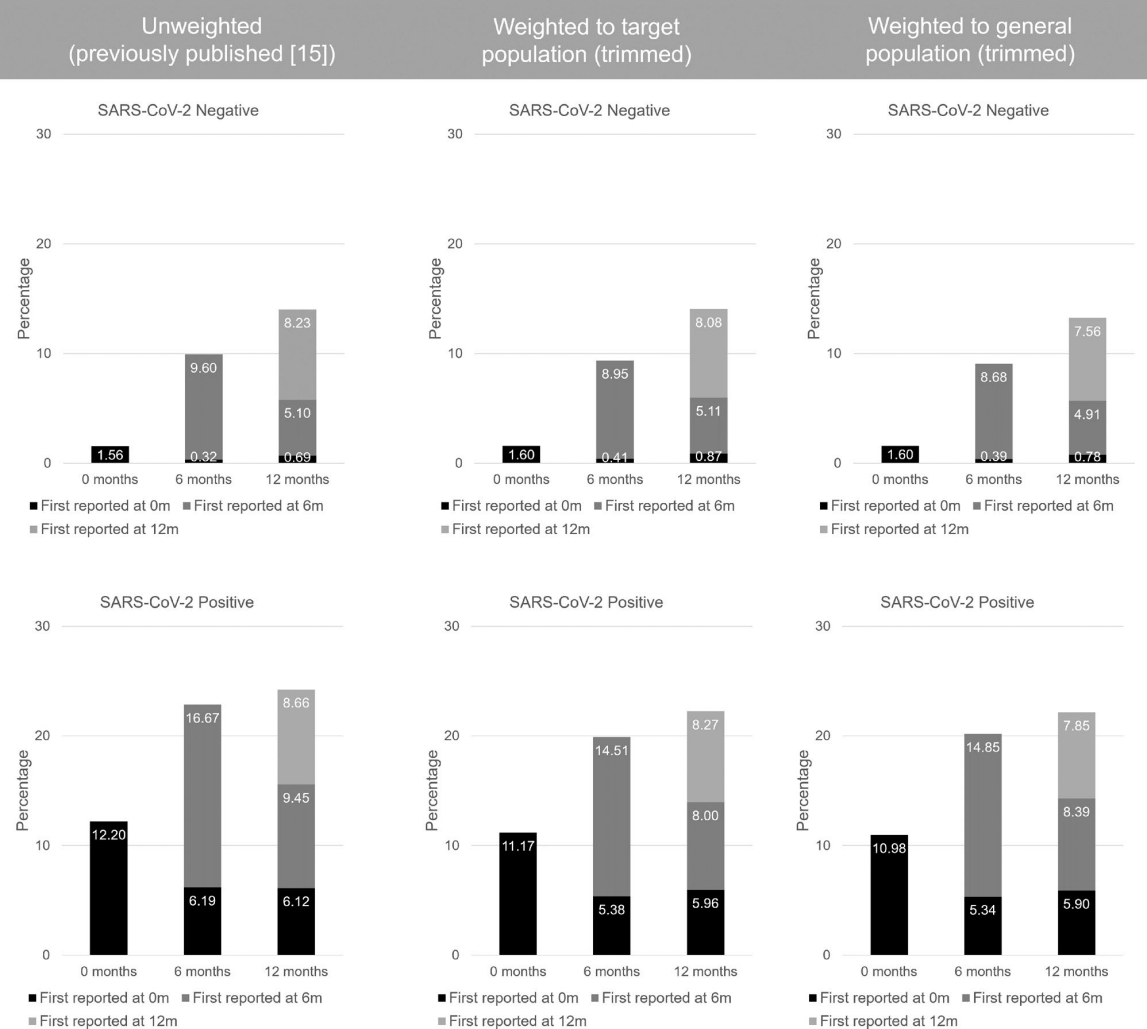
Weighted (trimmed) and unweighted shortness of breath prevalences 0-12-months post-index-PCR-test by time of first report

Consistent with published findings [15], the overall prevalence of tiredness and shortness of breath increased from baseline to 12-months post-index PCR-test in both test-positive and test-negative CYP even after weighting (and trimming) to the target and general populations (Tables 4 and 5; Figs. 6 and 7). For example, at time of testing, the unweighted overall prevalence of tiredness in CYP who tested negative for SARS-CoV-2 was 3.63%. When weighted (and trimmed) to the target population the prevalence was 3.51% and when weighted (and trimmed) to the general population the prevalence was 3.69% (Table 4). Likewise, prevalences of tiredness and shortness of breath by time of first report remained similar to published findings (Figs. 6 and 7). Results using trimmed and untrimmed weights were broadly similar (Additional File 1: Tables 2 and 3; Figs. 1 and 2). Table 4 (Additional File 1) shows the uncertainty around the generated target population weight (untrimmed); results are broadly consistent.

**Table 4:** Weighted and unweighted tiredness prevalences from baseline to 12 months post-index PCR-test

Timepoint	Unweighted (previously published [15])	Weighted to target population (trimmed)	Weighted to general population (trimmed)
Negatives			
0 months	3.63%	3.51%	3.69%
6 months	25.14%	23.44%	22.56%
12 months	33.41%	31.85%	31.14%
**Positives**			
0 months	27.19%	26.22%	25.41%
6 months	38.64%	38.28%	37.82%
12 months	45.89%	45.16%	44.67%

**Table 5:** Weighted and unweighted shortness of breath prevalences from baseline to 12 months post-index PCR-test

Timepoint	Unweighted (previously published [15])	Weighted to target population (trimmed)	Weighted to general population (trimmed)
Negatives			
0 months	1.56%	1.60%	1.60%
6 months	9.93%	9.36%	9.07%
12 months	14.02%	14.06%	13.25%
**Positives**			
0 months	12.20%	11.17%	10.98%
6 months	22.86%	19.89%	20.18%
12 months	24.24%	22.23%	22.14%

## Discussion

The present study aimed to (i) create weights for the CLoCk study at its data collection sweeps 3-, 6- and 12-months post-index PCR-test, and (ii) apply the developed survey weights to the analysis of shortness of breath and tiredness over the 12-month period to determine whether accounting for any biases in the target population, response, attrition or (re)infection affected published results. Flexible survey weights for the CLoCk study were developed and applied in an illustrative example. When applying the survey weights, results were consistent with published CLoCk findings [15]. That is, the overall prevalence of tiredness and shortness of breath increased over time from baseline to 12-months post-testing in both test-positive and test-negative CYP.

A major strength of the present study includes the flexibility of the survey weights developed whereby the creation of separate ‘mini’ survey weights (i.e., response, timely response and (re)infection) and the overall ‘envisioned population’ weight ensures researchers are able to combine them to re-create their specific target population, which will vary depending on the specific research question being asked. The interactive tool provided will allow researchers to re-calibrate their target population weights to the general population of CYP in England using the recent Census 2021 data. This re-calibration attempts to address the potential bias in the envisioned CLoCk population due to variation in PCR testing by region and stage of the pandemic [31, 32]. Furthermore, by trimming survey weights using a technique that is unaffected by the size of the largest survey weight [34], we improve the accuracy and precision of final parameter estimates in re-weighted analyses [37]. Moreover, we used a range of data from both the UKHSA dataset and the CLoCk questionnaire to develop the models that predicted the required conditional probabilities. We acknowledge that the *C* statistics, particularly for models used to predict the probability of responding given envisioned to take part and the probability of responding timely given responded were somewhat low ranging between 0.60 and 0.73. However, for the probability of responding given envisioned to take part, it should be noted that the *C* statistic cannot be further improved due to the lack of additional data relating to the envisioned CLoCk population (here, only data held on the UKHSA database for matching was available). Thus, for all survey weight generation, but here in particular, one should note the constraint deriving from the variables used to generate conditional probabilities and the potential for the non-response/attrition/selection mechanisms to be dependent on unmeasured variables. For example, it might be that those with severe tiredness are less likely to respond. Relatedly, our approach is appropriate when missingness is assumed to be dependent on observed characteristics, but as mentioned above this may not be the case. This is an important potential limitation, with the implication being survey weights do not fully adjust for such (non-response, attrition, and sample selection) bias, though we attempt to minimise its impact. In an attempt to avoid potential recall bias, for the latter two ‘mini’ weights, we made the pragmatic decision to only consider questionnaire data asked in relation to health and wellbeing at the time of questionnaire completion.

We acknowledge concerns regarding the use of stepwise selection processes whereby inclusion of too many candidate variables may result in nuisance variables being selected over true variables meaning the best model is not provided [38]. We were mindful of this when selecting the initial list of potential predictors, determined the best functional forms of continuous variables used in all regressions, and used theoretical arguments to inform our selection, as recommended [39]. Finally, it should be noted that the survey weights are estimated and if treated as observed there is a risk of overestimating the precision of the estimates. To address this, we provide an example of how variabilities due to generating the weights can be accounted for via bootstrapping.

## Conclusions

CLoCk is the largest known prospective study of Long Covid in non-hospitalised CYP, with over 30,000 respondents. Like all longitudinal population-based studies, issues regarding selection into the study and attrition over time need to be considered. The present findings suggest the CLoCk sample is representative of the envisioned and general populations of CYP in England, although the developed weights need to be utilised in multiple and different contexts to assess their impact and identify whether current conclusions are consistent across other CLoCk analyses. The same approach can and should be taken in other research studies to assess sample representativeness. Importantly, application of survey weights more generally is beneficial as a way of addressing the impact of potential bias.

### Electronic supplementary material

Below is the link to the electronic supplementary material.

Additional file 1. Additional Tables, Text and Figures. This file contains additional Tables 1, 2, 3 and 4, Text 1 and Figs. 1 and 2. Table 1. Further information on variables included in stepwise selection processes for weight generation and their handling. Text 1. Illustrative code demonstrating how uncertainty around generated weights can be accounted for via bootstrapping (with 1000 replications). Table 2. Tiredness prevalence 0 to 12-months post-index PCR-test weighted (trimmed and untrimmed) and unweighted. Table 3. Shortness of breath prevalence 0 to 12-months post-index PCR-test weighted (trimmed and untrimmed) and unweighted. Table 4. Illustrative example of tiredness prevalence 0 to 12-months post-index PCR-test weighted to the target population (untrimmed) with bootstrapped confidence intervals (1000 replications). Figure 1. Weighted (trimmed and untrimmed) and unweighted tiredness prevalences by time of first report. Figure 2. Weighted (trimmed and untrimmed) and unweighted shortness of breath prevalences by time of first report.

Additional file 2: Interactive online tool for re-calibration of survey weights to the general population. This can be used to re-calibrate final target population survey weights to the general population using data on sex, age, and region from the 2021 UK Census. The tool allows ratios of the Census data to CLoCk data reweighted to the target population to be produced and provides examples of what to do with these ratios.

## Data Availability

Data are not publicly available. All requests for data will be reviewed by the Children & young people with Long Covid (CLoCk) study team, to verify whether the request is subject to any intellectual property or confidentiality obligations. Requests for access to the participant-level data from this study can be submitted via email to the corresponding author with detailed proposals for approval. A signed data access agreement with the CLoCk team is required before accessing shared data.

## Abbreviations

UKHSA: United Kingdom Health Security Agency
UK: United Kingdom
CYP: Children and Young People
CLoCk: Children and young people with Long Covid
SDQ: Strengths and Difficulties Questionnaire
EQ-VAS: EuroQol Visual Analogue Scale
IMD: Index of Multiple Deprivation
CFS: Chalder Fatigue Scale
SWEMWS: Short Warwick Edinburgh Mental Wellbeing Scale
