# Supplementary material for: Developing survey weights to ensure representativeness in a national, matched cohort study: results from the children and young people with Long Covid (CLoCk) study
Source: BMC Med Res Methodol. 2024 Jun 20;24:134. doi: 10.1186/s12874-024-02219-0 (PMC11188173; doi:10.1186/s12874-024-02219-0)
Supplement: Supplementary file 1 — Additional file 1. Additional Tables, Text and Figures. This file contains additional Tables 1, 2, 3 and 4, Text 1 and Figs. 1 and 2. Table 1. Further information on variables included in stepwise selection processes for weight generation and their handling. Text 1. Illustrative code demonstrating how uncertainty around generated weights can be accounted for via bootstrapping (with 1000 replications). Table 2. Tiredness prevalence 0 to 12-months post-index PCR-test weighted (trimmed and untrimmed) and unweighted. Table 3. Shortness of breath prevalence 0 to 12-months post-index PCR-test weighted (trimmed and untrimmed) and unweighted. Table 4. Illustrative example of tiredness prevalence 0 to 12-months post-index PCR-test weighted to the target population (untrimmed) with bootstrapped confidence intervals (1000 replications). Figure 1. Weighted (trimmed and untrimmed) and unweighted tiredness prevalences by time of first report. Figure 2. Weighted (trimmed and untrimmed) and unweighted shortness of breath prevalences by time of first report. [file 12874_2024_2219_MOESM1_ESM.docx]

**Additional File 1:** **Additional Tables, Text and Figures**

**Table 1.** Further information on variables included in stepwise selection processes for weight generation and their handling

| Variables | Details | Collection point |
| --- | --- | --- |
| Index COVID Status | Assessed using UKHSA held data at the point of matching test-negatives and test-positives (Positive/Negative). | Matching |
| Age | Obtained from UKHSA at the point of matching test-negatives and test-positives. The functional form of this variable varies for each specific regression; see methods and Table 1 in main manuscript for details. | Matching |
| Sex | Obtained from UKHSA at the point of matching test-negatives and test-positives (Male/Female). | Matching |
| Region | Obtained from UKHSA at the point of matching test-negatives and test-positives (East Midlands; East of England; London; North East England; North West England; South East England; South West England; West Midlands; Yorkshire and the Humber). | Matching |
| IMD | Derived using CYP’s lower super output area (i.e., small local area level-based geographic hierarchy) where higher values were indicative of lower deprivation [1]. The functional form of this variable varies for each specific regression; see methods and Table 1 in main manuscript for details. | Matching |
| Ethnicity | Self-reported at enrolment, which can be 3-, 6-, or 12-months post-index PCR-test (Asian or Asian British; Black, African, or Caribbean; Mixed; White; Other; Prefer not to say). | 3, 6, 12 |
| Current number of symptoms | Derived from 21 symptoms which were mostly assessed as present/absent, ranging from no symptoms to 21 symptoms [2]. | 3, 6, 12 |
| Current health | Self-reported (I am not feeling quite right; I feel as healthy as normal). | 3, 6, 12 |
| Quality of life/functioning (EQ-5D-Y) | This scale measures problems (No/Some/A lot of problems) with (a) mobility, (b) self-care, (c) doing usual activities, (d) having pain or discomfort, and (e) feeling worried, sad or unhappy [3]. Included/excluded as a group of five variables with three categories each. | 3, 6, 12 |
| UCLA Loneliness Scale | The final loneliness score was derived as the sum of the three items in the UCLA Loneliness Scale whereby a score of eight and above denoted loneliness [4, 5]. | 3, 6, 12 |
| Current Loneliness | This is a direct measure of loneliness (“How often do you feel lonely”) which is scored 1-5 (Often/Always; Some of the time; Occasionally; Hardly Ever; Never) [4]. | 3, 6, 12 |
| Strengths and Difficulties Questionnaire (SDQ) | Five subscales (25 items) measure: (i) emotional symptoms, (ii) conduct problems, (iii) hyperactivity, (iv) peer relationships, and (v) prosocial skills. Each item was scored from 0 to 2, meaning total scores per subscale ranged from 0-10. A total difficulties score (ranging from 0-40) was derived as the sum of all but the prosocial subscale. The impact of difficulties on CYP is specified by the impact subscale, with scores ranging from 0-10 (higher scores denoting a higher impact).  The following cut-off points were used: ≥18 (total difficulties); ≥4 (peer difficulties); ≥5 (conduct problems); ≥6 (emotional symptoms); ≥7 (hyperactivity); ≤5 (prosocial skills) and ≥2 for impact [6]. | 3, 6, 12 |
| Short Warwick Edinburgh Mental Wellbeing Scale (SWEMWS) | Consists of seven items with a five-point scale (None of the time; Rarely; Some of the time; Often; All of the time) scored 1-5. Scores are summed to provide a mental wellbeing score ranging from 7-35. Poor wellbeing was characterised as a score of <19.6 [7] post-conversion to metric. | 3, 6, 12 |
| Chronic Fatigue (Chalder Fatigue Scale; CFQ-11) | Eleven items on a four point scale (Less than usual; No more than usual; More than usual; Much more than usual) coded as 0, 0, 1, 1. These were summed with scores ranging from 0-11 and fatigue defined as a score of four and above [8, 9]. | 3, 6, 12 |
| EuroQol Visual Analogue Scale (EQ-VAS) | 0% refers to the worst health imaginable and 100% to the best, scores went up in increments of 5 [10]. | 3, 6, 12 |
| Vaccination status | Self-reported at time of questionnaire completion (Yes/No) | 3, 6, 12 |

**Text 1.** Illustrative code demonstrating how uncertainty around generated weights can be accounted for via bootstrapping (with 1000 replications)

***********************

****create weights within the bootstrap programme****

***********************

capture program drop weight_ci

program define weight_ci, rclass

capture drop p_response_3m wt3mv3

capture drop p_response_6m response_wt6m

capture drop p_response_ontime_6m timely_response_wt6m

capture drop p_not_reinf_6m not_reinf_wt6m

capture drop wt6m

capture drop p_response_12m response_wt12m

capture drop p_response_ontime_12m timely_response_wt12m

capture drop p_not_reinf_12m not_reinf_wt12m

capture drop wt12m

capture drop wt_0_6_12m

preserve

***********************

*3m

***********************

*******************************

****P(Responding | invited)****

*******************************

logistic response_3m ib1.PHE_sex##c.PHE_age3 ib1.PHE_sex##c.PHE_age3ln ib1.PHE_sex##ib1.Region ib1.PHE_sex##ib0.PHE_result ib1.PHE_sex##c.IMD_c ib1.PHE_sex##c.IMD_sq c.PHE_age3##ib1.Region c.PHE_age3ln##ib1.Region c.PHE_age3##ib0.PHE_result c.PHE_age3ln##ib0.PHE_result c.PHE_age3##c.IMD_c c.PHE_age3##c.IMD_sq c.PHE_age3ln##c.IMD_c c.PHE_age3ln##c.IMD_sq ib1.Region##ib0.PHE_result ib1.Region##c.IMD_c ib1.Region##c.IMD_sq ib0.PHE_result##c.IMD_c ib0.PHE_result##c.IMD_sq c.PHE_age3##c.PHE_age3ln c.IMD_sq##c.IMD_c if invited_3m==1

predict p_response_3m if invited_3m==1

label var p_response_3m "p(responded at 3m given invited at 3m)"

gen response_wt3m = 1/p_response_3m if invited_3m==1

****************************************

****P(Responding timely | responded)****

****************************************

logistic response_ontime_3m ib1.PHE_sex ib1.PHE_age3cat ib1.Region ib0.PHE_result c.IMD_sq c.IMD_c ib1.vaccine_3m ib2.phealth_curr_3m ib2.lonely_curr_3m if invited_3m==1 & response_3m==1

predict p_response_ontime_3m if invited_3m==1 & response_3m==1

label var p_response_ontime_3m "p(responded timely at 3m given responded at 3m)"

gen timely_response_wt3m = 1/p_response_ontime_3m if invited_3m==1 & response_3m==1

**************************************************

******P(Not (Re)infected | responded timely)******

**************************************************

logistic not_reinf_3m ib1.PHE_sex c.PHE_age c.IMD_sq c.IMD_c c.swemwbs_metric3m ib2.phealth_curr_3m c.EQVAS_curr_3m c.curr_total_3m if response_ontime_3m==1 & response_3m==1 & invited_3m==1

predict p_not_reinf_3m if invited_3m==1 & response_3m==1 & response_ontime_3m==1

label var p_not_reinf_3m "p(not (re)infected given responded timely at 3m)"

gen not_reinf_wt3m = 1/p_not_reinf_3m if invited_3m==1 & response_3m==1 & response_ontime_3m

****************************************

****Base 'invited population' Weight****

****************************************

gen wt3m=not_reinf_wt3m*timely_response_wt3m*response_wt3m if invited_3m==1 & response_3m==1 & response_ontime_3m==1 & not_reinf_3m==1

***********************

*6m

***********************

*******************************

****P(Responding | invited)****

*******************************

logistic response_6m ib1.PHE_sex##c.PHE_age3 ib1.PHE_sex##c.PHE_age3ln ib1.PHE_sex##ib1.Region ib1.PHE_sex##ib0.PHE_result ib1.PHE_sex##c.IMD_c ib1.PHE_sex##c.IMD_sq c.PHE_age3##ib1.Region c.PHE_age3ln##ib1.Region c.PHE_age3##ib0.PHE_result c.PHE_age3ln##ib0.PHE_result c.PHE_age3##c.IMD_c c.PHE_age3##c.IMD_sq c.PHE_age3ln##c.IMD_c c.PHE_age3ln##c.IMD_sq ib1.Region##ib0.PHE_result ib1.Region##c.IMD_c ib1.Region##c.IMD_sq ib0.PHE_result##c.IMD_c ib0.PHE_result##c.IMD_sq c.PHE_age3##c.PHE_age3ln c.IMD_c##c.IMD_sq if invited_6m==1

predict p_response_6m if invited_6m==1

label var p_response_6m "p(responded at 6m given invited at 6m)"

gen response_wt6m = 1/p_response_6m if invited_6m==1

****************************************

****P(Responding timely | responded)****

****************************************

logistic response_ontime_6m ib1.PHE_sex ib1.PHE_age3cat ib1.Region ib0.PHE_result c.IMD_2 c.IMD_sqrt ib6.eth6m c.EQVAS_curr_6m c.curr_total_6m ib1.vaccine_6m if invited_6m==1 & response_6m==1

predict p_response_ontime_6m if invited_6m==1 & response_6m==1

label var p_response_ontime_6m "p(responded timely at 6m given responded at 6m)"

gen timely_response_wt6m = 1/p_response_ontime_6m if invited_6m==1 & response_6m==1

**********************************************

****P(Not (Re)infected | responded timely)****

**********************************************

logistic not_reinf_6m ib1.PHE_sex c.PHE_age3 c.PHE_age3ln ib1.Region ib0.PHE_result c.IMD_sq c.IMD_c ib1.vaccine_6m c.curr_total_6m c.SDQ6m ib1.eq5_curr_mobility6m ib1.eq5_curr_look6m ib1.eq5_curr_usual6m ib1.eq5_curr_pain6m ib1.eq5_curr_sad6m if response_ontime_6m==1 & response_6m==1 & invited_6m==1

predict p_not_reinf_6m if invited_6m==1 & response_6m==1 & response_ontime_6m==1

label var p_not_reinf_6m "p(not (re)infected given responded timely at 6m)"

gen not_reinf_wt6m = 1/p_not_reinf_6m if invited_6m==1 & response_6m==1 & response_ontime_6m

****************************************

****Base 'invited population' Weight****

****************************************

gen wt6m=not_reinf_wt6m*timely_response_wt6m*response_wt6m if invited_6m==1 & response_6m==1 & response_ontime_6m==1 & not_reinf_6m==1

***********************

*12m

***********************

***********************************

****P(Responding given invited)****

***********************************

logistic response_12m ib1.PHE_sex##c.PHE_age3 ib1.PHE_sex##c.PHE_age3ln ib1.PHE_sex##ib1.Region ib1.PHE_sex##ib0.PHE_result ib1.PHE_sex##c.IMD_05 c.PHE_age3##ib1.Region c.PHE_age3ln##ib1.Region c.PHE_age3##ib0.PHE_result c.PHE_age3ln##ib0.PHE_result c.PHE_age3##c.IMD_05 c.PHE_age3ln##c.IMD_05 ib1.Region##ib0.PHE_result ib1.Region##c.IMD_05 ib0.PHE_result##c.IMD_05 c.PHE_age3##c.PHE_age3ln if invited_12m==1

predict p_response_12m if invited_12m==1

label var p_response_12m "p(responded at 12m given invited at 12m)"

gen response_wt12m = 1/p_response_12m if invited_12m==1

****************************************

****P(Responding timely | responded)****

****************************************

logistic response_ontime_12m ib1.PHE_sex c.PHE_age ib1.Region ib0.PHE_result c.IMD_c c.IMD_sq ib6.eth12m ib1.vaccine_12m c.cfs_likert12m if invited_12m==1 & response_12m==1

predict p_response_ontime_12m if invited_12m==1 & response_12m==1

label var p_response_ontime_12m "p(responded timely at 12m given responded at 12m)"

gen timely_response_wt12m = 1/p_response_ontime_12m if invited_12m==1 & response_12m==1

**********************************************

****P(Not (Re)infected | responded timely)****

**********************************************

logistic not_reinf_12m ib1.PHE_sex c.PHE_age ib1.Region ib0.PHE_result c.IMD_neg2 c.IMD_pos2 ib6.eth12m c.curr_total_12m c.SDQ12m ib1.eq5_curr_mobility12m ib1.eq5_curr_look12m ib1.eq5_curr_usual12m ib1.eq5_curr_pain12m ib1.eq5_curr_sad12m ib2.phealth_curr_12m c.EQVAS_curr_12m ib2.lonely_curr_12m ib1.vaccine_12m if response_ontime_12m==1 & response_12m==1 & invited_12m==1

predict p_not_reinf_12m if invited_12m==1 & response_12m==1 & response_ontime_12m==1

label var p_not_reinf_12m "p(not (re)infected given responded timely at 12m)"

gen not_reinf_wt12m = 1/p_not_reinf_12m if invited_12m==1 & response_12m==1 & response_ontime_12m

****************************************

****Base 'invited population' Weight****

****************************************

gen wt12m=not_reinf_wt12m*timely_response_wt12m*response_wt12m if invited_12m==1 & response_12m==1 & response_ontime_12m==1 & not_reinf_12m==1

***********************

**** weights generated now need to be combined

***********************

gen wt_0_6_12m = .

replace wt_0_6_12m = response_wt3m*wt6m*wt12m if not_reinf_6m==1 & not_reinf_12m==1 //Jan, Feb, March

replace wt_0_6_12m = wt6m*wt12m if not_reinf_6m==1 & not_reinf_12m==1 & not_reinf_3mv2==. & not_reinf_3mv2!=0 //Oct, Nov, Dec

*calcuate the % of tiredneess in -ves and +ves at 0, 6 and 12m

forval num = 0(6)12 {

tab tired_`num'm if PHE_result==0 & not_reinf_6m==1 & not_reinf_12m==1 [iw=wt_0_6_12m ], mi matcell(table)

local tired`num'_neg = 100*table[2,1]/r(N)

tab tired_`num'm if PHE_result==1 & not_reinf_6m==1 & not_reinf_12m==1 [iw=wt_0_6_12m ], mi matcell(table)

local tired`num'_pos = 100*table[2,1]/r(N)

}

return scalar tired0_neg = `tired0_neg'

return scalar tired6_neg = `tired6_neg'

return scalar tired12_neg = `tired12_neg'

return scalar tired0_pos = `tired0_pos'

return scalar tired6_pos = `tired6_pos'

return scalar tired12_pos = `tired12_pos'

restore

end

bootstrap r(tired0_neg) r(tired6_neg) r(tired12_neg) r(tired0_pos) r(tired6_pos) r(tired12_pos), reps(1000) nodrop : weight_ci

**Table 2.** Tiredness prevalence 0 to 12-months post-index PCR-test weighted (trimmed and untrimmed) and unweighted

| **Timepoint** | **Unweighted**  **(previously published [11])** | **Weighted to target population (untrimmed)** | **Weighted to target population (trimmed)** | **Weighted to general population**  **(untrimmed)** | **Weighted to general population**  **(trimmed)** |
| --- | --- | --- | --- | --- | --- |
|  |  |  |  |  |  |
| **Negatives** |  |  |  |  |  |
|  |  |  |  |  |  |
| 0 months | 3.63% | 6.88% | 3.51% | 5.71% | 3.69% |
| 6 months | 25.14% | 22.92% | 23.44% | 23.55% | 22.56% |
| 12 months | 33.41% | 29.94% | 31.85% | 30.70% | 31.14% |
|  |  |  |  |  |  |
| **Positives** |  |  |  |  |  |
|  |  |  |  |  |  |
| 0 months | 27.19% | 25.19% | 26.22% | 24.54% | 25.41% |
| 6 months | 38.64% | 40.23% | 38.28% | 38.40% | 37.82% |
| 12 months | 45.89% | 44.48% | 45.16% | 43.33% | 44.67% |
|  |  |  |  |  |  |

**Table 3.** Shortness of breath prevalence 0 to 12-months post-index PCR-test weighted (trimmed and untrimmed) and unweighted

| **Timepoint** | **Unweighted**  **(previously published [11])** | **Weighted to target population (untrimmed)** | **Weighted to target population (trimmed)** | **Weighted to general population**  **(untrimmed)** | **Weighted to general population**  **(trimmed)** |
| --- | --- | --- | --- | --- | --- |
|  |  |  |  |  |  |
| **Negatives** |  |  |  |  |  |
|  |  |  |  |  |  |
| 0 months | 1.56% | 3.18% | 1.60% | 2.53% | 1.60% |
| 6 months | 9.93% | 8.46% | 9.36% | 8.68% | 9.07% |
| 12 months | 14.02% | 14.11% | 14.06% | 15.08% | 13.25% |
|  |  |  |  |  |  |
| **Positives** |  |  |  |  |  |
|  |  |  |  |  |  |
| 0 months | 12.20% | 12.10% | 11.17% | 11.68% | 10.98% |
| 6 months | 22.86% | 20.28% | 19.89% | 19.34% | 20.18% |
| 12 months | 24.24% | 20.74% | 22.23% | 20.68% | 22.14% |
|  |  |  |  |  |  |

**Table 4.** Illustrative example of tiredness prevalence 0 to 12-months post-index PCR-test weighted to the target population (untrimmed) with bootstrapped confidence intervals (1000 replications)

| **Timepoint** | **Unweighted**  **(previously published [11])** | **Weighted to target population (untrimmed)** |
| --- | --- | --- |
|  |  |  |
| **Negatives** |  |  |
|  |  |  |
| 0 months | 3.63% | 6.88% (3.22,10.53) |
| 6 months | 25.14% | 22.92% (18.53,27.31) |
| 12 months | 33.41% | 29.94% (25.34,34.54) |
|  |  |  |
| **Positives** |  |  |
|  |  |  |
| 0 months | 27.19% | 25.19% (20.77,29.62) |
| 6 months | 38.64% | 40.23**%** (34.85,45.61) |
| 12 months | 45.89% | 44.48% (39.08,49.88) |
|  |  |  |

**Figure 1.** Weighted (trimmed and untrimmed) and unweighted tiredness prevalences by time of first report


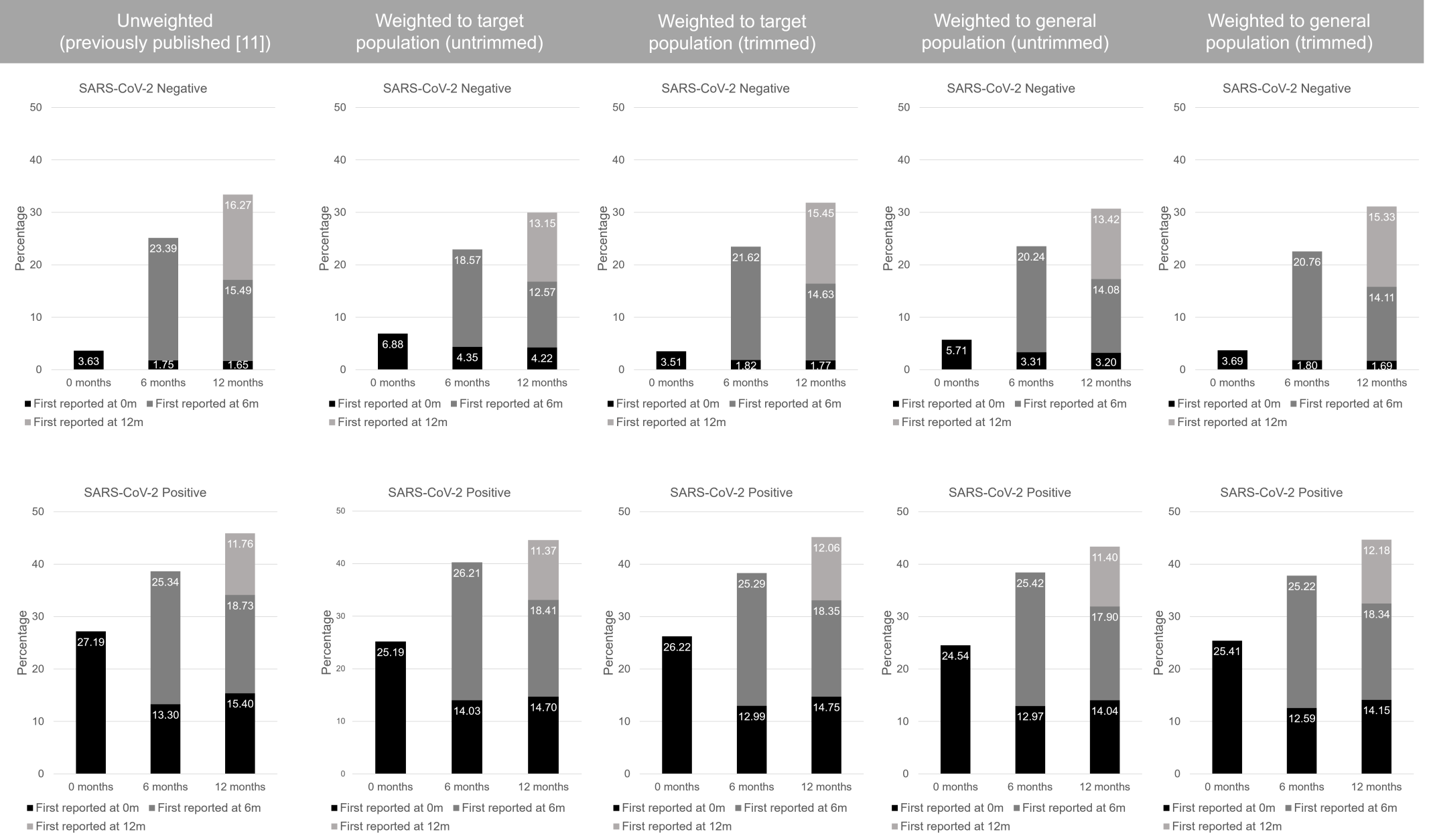


**Figure 2.** Weighted (trimmed and untrimmed) and unweighted shortness of breath prevalences by time of first report


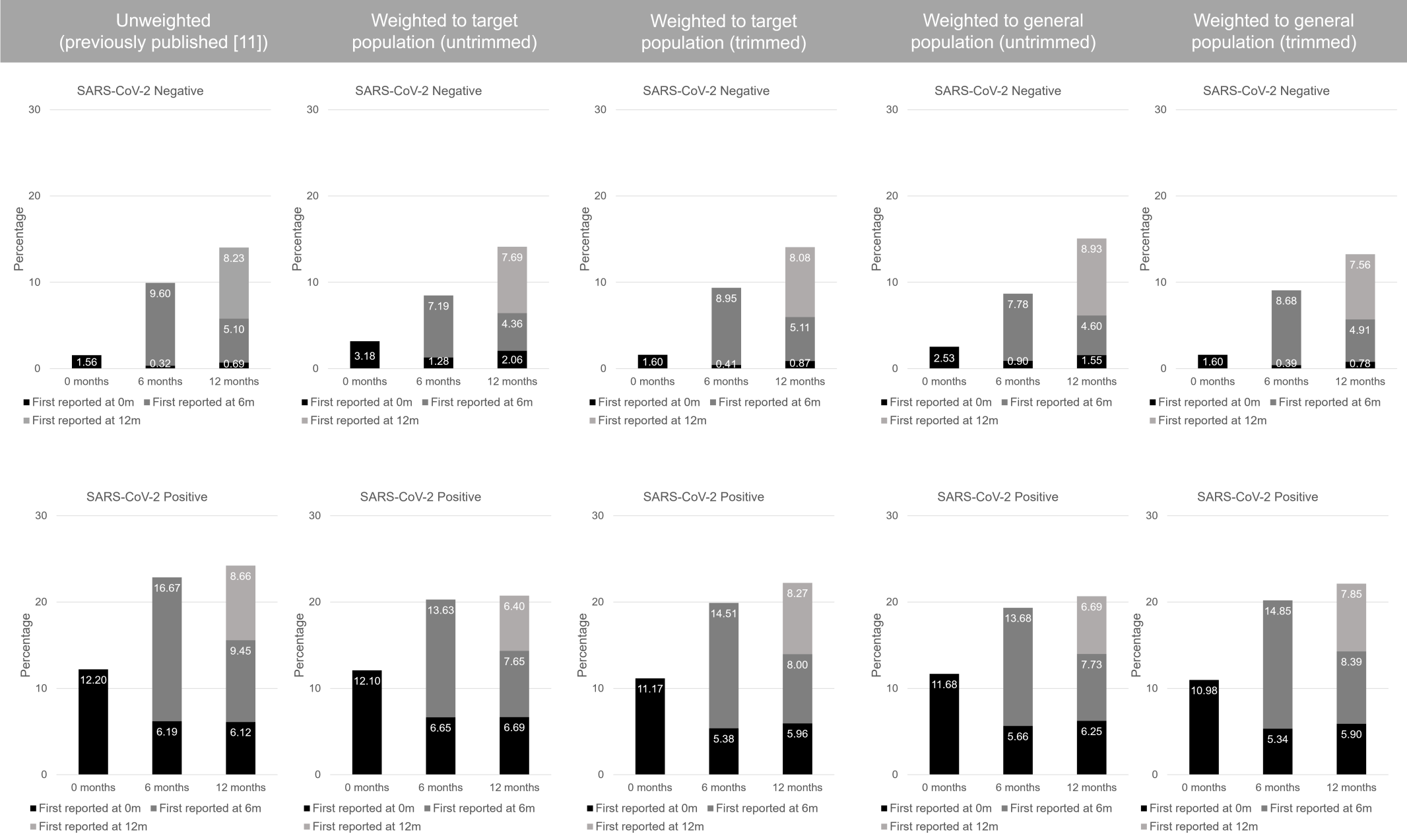


**References**

1. Ministry of Housing Communities & Local Government. English indices of deprivation 2015 2015 [Available from: <https://www.gov.uk/government/statistics/english-indices-of-deprivation-2015>.

2. Stephenson T, Pinto Pereira SM, Shafran R, de Stavola BL, Rojas N, McOwat K, et al. Physical and mental health 3 months after SARS-CoV-2 infection (long COVID) among adolescents in England (CLoCk): a national matched cohort study. The Lancet Child & Adolescent Health. 2022;6(4):230-9.

3. Wille N, Badia X, Bonsel G, Burström K, Cavrini G, Devlin N, et al. Development of the EQ-5D-Y: a child-friendly version of the EQ-5D. Qual Life Res. 2010;19(6):875-86.

4. Office for National Statistics. Measuring loneliness: guidance for use of the national indicators on surveys 2018 [Available from: <https://www.ons.gov.uk/peoplepopulationandcommunity/wellbeing/methodologies/measuringlonelinessguidanceforuseofthenationalindicatorsonsurveys#recommended-measures-for-children>.

5. Klein EM, Zenger M, Tibubos AN, Ernst M, Reiner I, Schmalbach B, et al. Loneliness and its relation to mental health in the general population: Validation and norm values of a brief measure. Journal of Affective Disorders Reports. 2021;4:100120.

6. youthinmind. Information for researchers and professionals about the Strengths & Difficulties Questionnaires 2022 [Available from: <https://www.sdqinfo.org/>.

7. Ng Fat L, Scholes S, Boniface S, Mindell J, Stewart-Brown S. Evaluating and establishing national norms for mental wellbeing using the short Warwick-Edinburgh Mental Well-being Scale (SWEMWBS): findings from the Health Survey for England. Qual Life Res. 2017;26(5):1129-44.

8. Chalder T, Berelowitz G, Pawlikowska T, Watts L, Wessely S, Wright D, et al. Development of a fatigue scale. J Psychosom Res. 1993;37(2):147-53.

9. Loge JH, Ekeberg O, Kaasa S. Fatigue in the general Norwegian population: normative data and associations. J Psychosom Res. 1998;45(1):53-65.

10. Feng Y, Parkin D, Devlin NJ. Assessing the performance of the EQ-VAS in the NHS PROMs programme. Qual Life Res. 2014;23(3):977-89.

11. Pinto Pereira SM, Shafran R, Nugawela MD, Panagi L, Hargreaves D, Ladhani SN, et al. Natural course of health and well-being in non-hospitalised children and young people after testing for SARS-CoV-2: A prospective follow-up study over 12 months. The Lancet Regional Health – Europe. 2022.
